# Single-wall carbon nanotubes improve cell survival rate and reduce oxidative injury in cryopreservation of *Agapanthus praecox* embryogenic callus

**DOI:** 10.1186/s13007-020-00674-6

**Published:** 2020-09-21

**Authors:** Li Ren, Shan Deng, Yunxia Chu, Yiying Zhang, Hong Zhao, Hairong Chen, Di Zhang

**Affiliations:** 1 Institute for Agri-food Standards and Testing Technology, Shanghai Academy of Agricultural Sciences, NO. 888, Rd. Yezhuang, Shanghai, 201403 China; 2 Department of Landscape Science and Engineering, School of Design, Shanghai Jiao Tong University, NO. 800, Rd. Dong Chuan, Shanghai, 200240 China

**Keywords:** *Agapanthus praecox*, Cryopreservation, Oxidative stress, Single-wall carbon nanotubes

## Abstract

**Background:**

Cryopreservation is the best way for long-term in vitro preservation of plant germplasm resources. The preliminary studies found that reactive oxygen species (ROS) induced oxidative stress and ice-induced membrane damage are the fundamental causes of cell death in cryopreserved samples. How to improve plant cryopreservation survival rate is an important scientific issue in the cryobiology field.

**Results:**

This study found that the survival rate was significantly improved by adding single-wall carbon nanotubes (SWCNTs) to plant vitrification solution (PVS) in cryopreservation of *Agapanthus praecox* embryogenic callus (EC), and analyzed the oxidative response of cells during the control and SWCNTs-added cryopreservation protocol. The SWCNTs entered EC at the step of dehydration and mainly located around the cell wall and in the vesicles, and most of SWCNTs moved out of EC during the dilution step. Combination with physiological index and gene quantitative expression results, SWCNTs affect the ROS signal transduction and antioxidant system response during plant cryopreservation. The EC treated by SWCNTs had higher antioxidant levels, like POD, CAT, and GSH than the control group EC. The EC mainly depended on the AsA-GSH and GPX cycle to scavenge H_2_O_2_ in the control cryopreservation, but depended on CAT in the SWCNTs-added cryopreservation which lead to low levels of H_2_O_2_ and MDA. The elevated antioxidant level in dehydration by adding SWCNTs enhanced cells resistance to injury during cryopreservation. The ROS signals of EC were balanced and stable in the SWCNTs-added cryopreservation.

**Conclusions:**

The SWCNTs regulated oxidative stress responses of EC during the process and controlled oxidative damages by the maintenance of ROS homeostasis to achieve a high survival rate after cryopreservation. This study is the first to systematically describe the role of carbon nanomaterial in the regulation of plant oxidative stress response, and provided a novel insight into the application of nanomaterials in the field of cryobiology.

## Background

The long-term preservation of plants in vitro cultures of cell cultures, embryogenic materials, and endangered germplasm is a vital requirement around the world [1]. Cryopreservation, the storage of living cells, tissues, organs or whole plants in extra low temperature, such as in liquid nitrogen (LN), is thought to be the ideal method to realize the safe and cost-efficient medium- and long-term preservation of almost all economically important crops [2–4]. Vitrification-based cryopreservation has been successfully applied to long-term preservation by achieving the glassy state in cryopreservation of many plant species for its rapid and convenient procedure [5]. Plant vitrification solution (PVS), a commonly used cryoprotectant in vitrification-based cryopreservation, can replace cellular water, alter the freezing behavior, and prevent ultra-water loss [6].

Vitrification-based cryopreservation achieves the storage by enhancing cell viscosity, and accompanies many stresses leading to the accumulation of reactive oxygen species (ROS) [7]. ROS-induced oxidative stress is the fundamental trigger of cell death during cryopreservation [8]. Ren et al. [7, 9] utilized comparative transcriptomics to obtain genes responses to cryoinjury and revealed that peroxidation was a key element affecting viability, and genes related to oxidative stress played important roles in *Arabidopsis thaliana* cryopreservation. Zhang et al. [10] revealed that the oxidative stress and apoptosis were the major factors that injure embryogenic callus (EC) during *Agapanthus praecox* cryopreservation.

Plants have complex antioxidant systems including antioxidant enzymes, such as catalase (CAT), superoxide dismutase (SOD), glutathione reductase (GR) and ascorbate peroxidase (APX), and non-enzymatic antioxidants, like glutathione (GSH) and ascorbic acid (AsA) [11]. The high antioxidant level is related to the tolerance to cryopreservation [12]. In antioxidant enzymes, SOD removes O_2_^−^ to H_2_O_2_, and CAT catalyzes H_2_O_2_ to O_2_ and H_2_O [13, 14]. In non-enzymatic antioxidants, AsA and GSH helps keep a normal REDOX state to counteract oxidative damages [15].

Many researchers have been focusing on the improvement of the cryopreservation procedure by adding exogenous compounds to the cryoprotectant to reduce damages. Nanomaterials have a good effect to improve the cryoprotectant, due to its small particle size and large specific surface [16]. After adding nanomaterials to the cryoprotectant can enhance the thermal conductivity and relative viscosity, promote vitrification, improve the stability of solution during the rewarming process, and suppress the occurrence of devitrification [17–19]. Han et al. [20] added 0.2% Nano-diamond to the ethylene glycol cryoprotectant, and found it doubled the freezing rate and decreased the glass transition temperature significantly. In addition, adding 0.05% hydroxyapatite to the cryoprotectant can improve the pig oocytes developmental rate from 14.7% to 30.4% after cryopreservation [19, 21]. After adding Nanomaterials Rhodiola Sachanensis Polysaccharide (NRSP), the motility, acrosome integrity and membrane integrity of boar sperm were improved significantly compared to the control groups after freezing and thawing [22]. With the addition of NRSP in the cryoprotectant, the malondialdehyde (MDA) content decreased and the activity of SOD increased [21]. Furthermore, lecithin nanoparticles can enhance the cryosurvival of caprine sperm [23], and albumin coated copper-cysteine nanozyme and Au–Ag-AFT nanozyme both can improve survival of human sperm after cryopreservation by reducing oxidative stress [24, 25]. Enrichment of semen extender with selenium nanoparticles improved fertility rate through decreasing peroxidation and injury during cryopreservation of Holstein bulls [26].

However, some nanomaterials have been reported to induce cell death [17, 18], and carbon nanomaterials (CNMs) have better biosafety and biocompatibility [27, 28]. 60-h seedlings of *Arabidopsis thaliana* are often used as an experimental model to evaluate the optimal effect of exogenous additives on cryopreservation of plants [7]. In our previous studies, CNMs including graphene, single-walled carbon nanotubes (SWCNTs) and graphene quantum dots have been applied in this cryopreservation model to identify their effects on cryopreservation, and 0.1 g/L SWCNTs in PVS2 was the most effective one (unpublished results). Then we applied it to the callus or protocorm cryopreservation of *lily, Cymbidium* and *Anoectochilu*, and it also played a positive role in each process. Chen et al. [28] investigated the efficiency of SWCNTs, Graphene, Graphene quantum dots and fullerene (C_60_) on the cryopreservation of *Agapanthus praecox* callus by adding to PVS2. 0.3 g/L fullerene and 0.1 g/L SWCNTs were the best two CNMs. The effect on vitrification behaviors was detected, and the glass transition temperatures showed no significant difference on Nano-PVS2. Raman spectroscopy and transmission electron microscopy (TEM) analyses demonstrated that the SWCNTs and fullerene were able to enter the callus cells and protect the cell structure [28].

*Agapanthus praecox* is a perennial herbaceous plant from Agapanthaceae family and is endemic to southern Africa [29, 30]. *A. praecox* is known for its ornamental and medicinal values. It is popular as the potted plant, cut flower, and landscape plant due to its multiflorous character, long flowering duration, and wide spectrum of colors [30, 31]. It contains various bioactive compounds such as saponins and chalconoids, which possess anti-inflammatory, antitussive, immunoregulatory, and antibacterial properties [32]. Some rapid micropropagation protocols for clonal propagation and conservation purposes were reported including the induction of EC [33–35]. The EC is an important germplasm resource, but somatic embryogenic ability is often lost during long-term subculture. Cryopreservation is an effective way to solve this problem. Generally, seeds, with characteristic dense cytoplasm and low water content, are easily cryopreserved by vitrification. However, callus cultures, suspension cultures, shoot tips, and recalcitrant seeds are more difficult to cryopreserve due to their higher water content, active metabolic activities, complex biological processes, and sensitive stress responses [7, 10]. *A. praecox* EC is representatively cryoinjury-susceptible materials for its high moisture content and low-temperature sensitivity [30]. In previous, we have initially cryopreserved the EC of *A. praecox*. In this study, SWCNTs adding into PVS2 evaluated the survival rate of EC after cryopreservation. It aims to point the positive effects to oxidative stress in cryopreservation, and provides a new application of CNMs using as protectants during cryopreservation. This study is the first time to systematically investigate the effects of nanomaterial to regulate cell responses to oxidative stress in plant cryopreservation applications.

## Results

### Effects of SWCNTs on the cell viability after cryopreservation

**Fig. 1:**
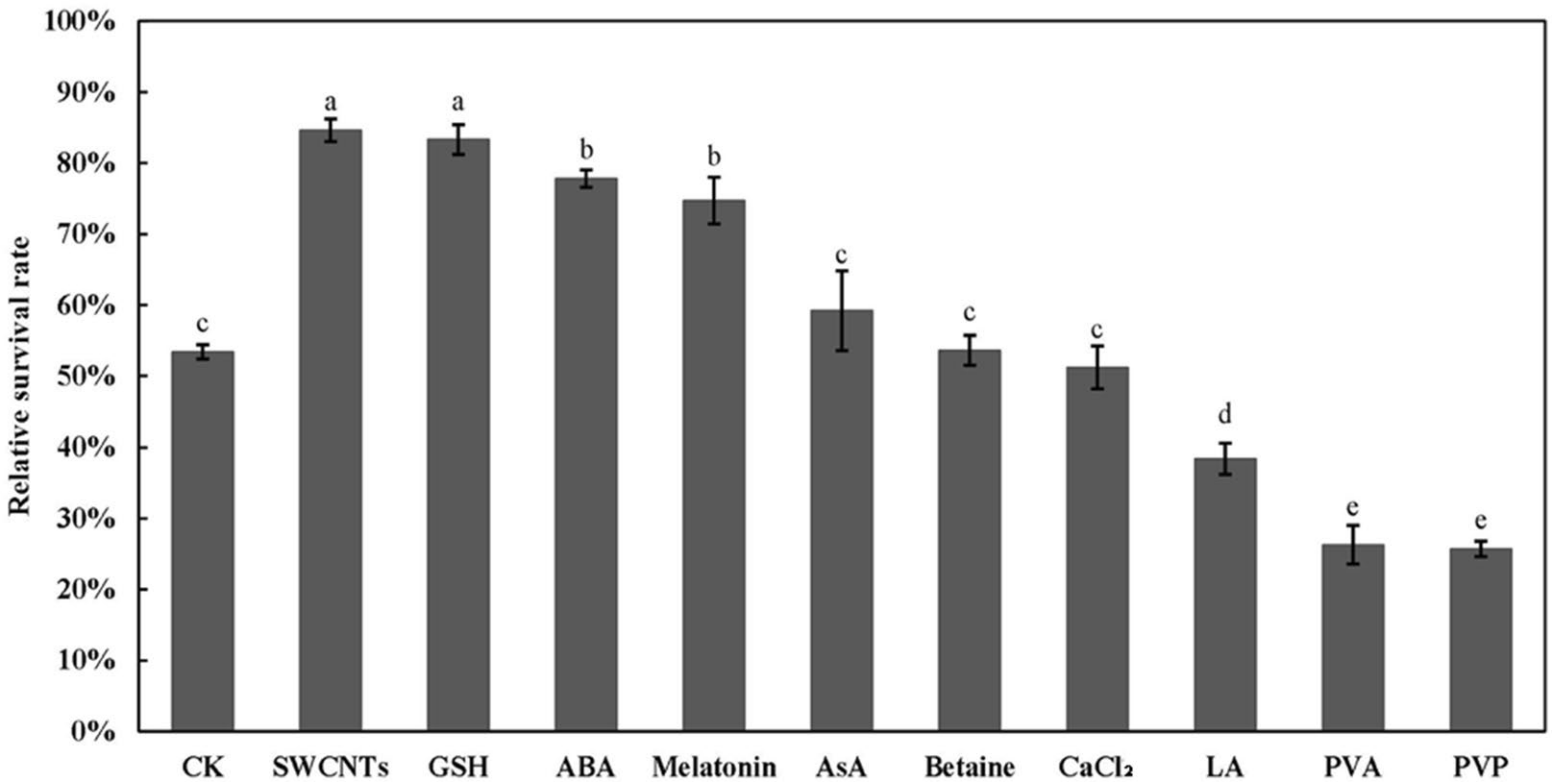
Effects of different exogenous substances on the survival rate of *Agapanthus praecox* EC cryopreservation. Concentration of exogenous substances added are: 0.1 g/L SWCNTs, 0.08 mM GSH, 1 μM ABA, 0.1 μM melatonin, 1 mM AsA, 10 mM betaine, 1 mM CaCl_2_, 6 mM LA, 6 mM PVA, 3% PVP. Values with different small letters are significantly different between different treatment at 0.05 level

Based on the previous studies, CNMs have been applied in the *Arabidopsis* cryopreservation model to identify their effects on cryopreservation, and 0.1 g/L SWCNTs in PVS2 was the most effective one (unpublished results). In this study, we applied it to the EC cryopreservation of *A. praecox*, and 0.1 g/L SWCNTs have improved the relative survival rate from 53.42% to 84.57% (Fig. 1). Comparing it to the effects of other compounds adding to PVS2 in previous studies including 0.08 mM GSH, 1 μM abscisic acid (ABA), 0.1 μM melatonin, 1 mM AsA, 10 mM betaine, 1 mM CaCl_2_, 6 mM lipoic acid (LA), 6 mM polyvinyl alcohol (PVA), or 3% polyvinyl pyrrolidone (PVP), 0.1 g/L SWCNTs had a significant effect on the EC survival increasing by 58.31% higher than others (Fig. 1).

### Localizations of SWCNTs in EC during cryopreservation

**Fig. 2:**
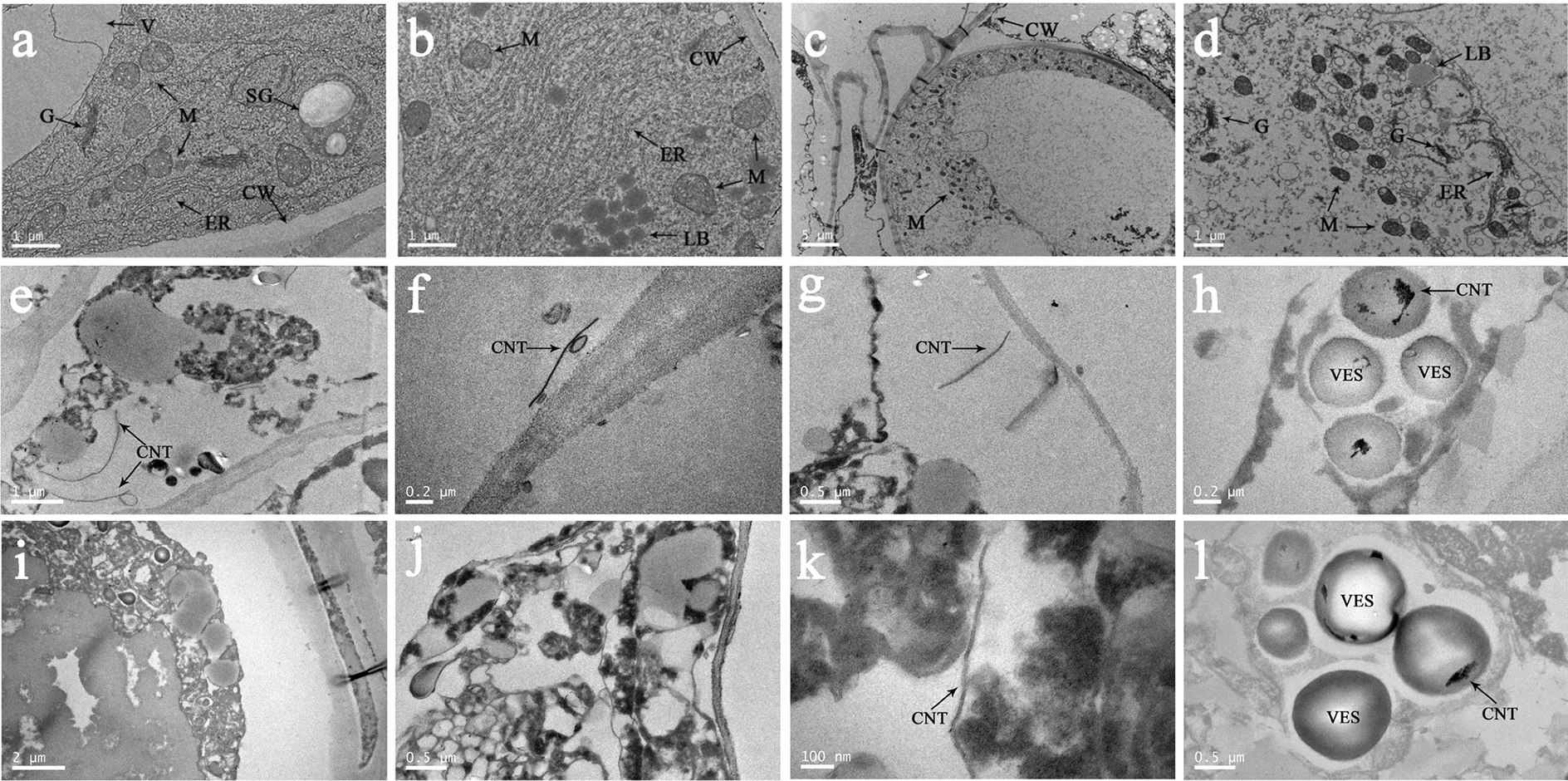
Cell ultrastructure observation of *Agapanthus praecox* during the SWCNTs-added cryopreservation. **a**, **b** the EC before cryopreservation, **c**, **d** the EC after pre-culture, **e**–**h** the EC after dehydration, **i**–**l** the EC after dilution. Cell wall (CW), endoplasmic reticulum (ER), golgi apparatus (G), lipid body (LB), mitochondrion (M), starch grains (SG), vesicle (VES)

Transmission electron microscopy imaging provided accurate results, which displayed the cellular status of EC and localizations of SWCNTs inside the EC. Many mitochondria, Golgi apparatus, and endoplasmic reticulum distributed in the EC with complete and compact cell structure before cryopreservation (Fig. 2a, b). After pre-culture, mitochondria, and cell membrane exhibited slight contraction in EC (Fig. 2c, d). The SWCNTs entered EC at the step of dehydration, and mainly located around the cell wall and in the vesicles (Fig. 2e–h). It is demonstrated that plasmolysis, protoplast concentrated, organelles damage and more intracellular vesicles were observed after dehydration (Fig. 2h). The dilution treatment caused most of SWCNTs to move out of EC, and the retained SWCNTs were mostly tube-like fragmented, mainly distributed in the cytoplasm and vesicles (Fig. 2i–l).

### Effects of SWCNTs on the ROS and MDA contents during cryopreservation

**Fig. 3:**
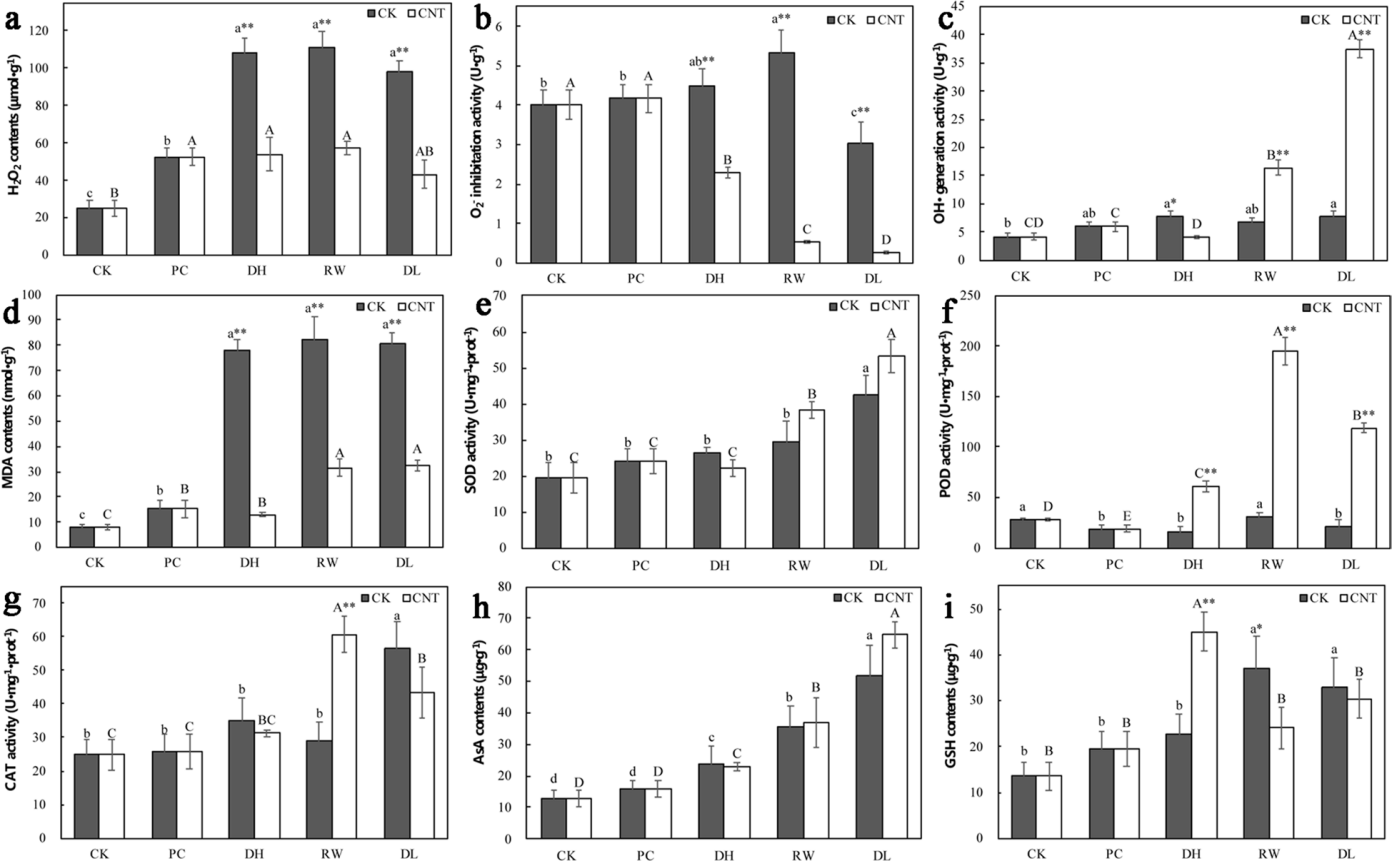
Physiological indices detection of SWCNTs added in cryopreservation system. Physiological indices were determined with at least three repeats. Bars represent means and standard deviation over triplicate detection. Values with different lowercase letters are significantly different among samples in the control group at 0.05 level. Values with different uppercase letters are significantly different among samples in the SWCNTs group at 0.05 level. * are significantly different between different groups in the same stage at 0.05 level, and ** are highly significantly different between different groups in the same stage at 0.01 level. The CK group is the cryopreservation without adding SWCNTs, and the CNT group is the SWCNTs-added cryopreservation. CK, untreated; PC, after pre-culture; DH, after dehydration; RW, after rapid cooling-warming; DL, after dilution

In the control procedure, H_2_O_2_ contents highly increased in dehydration and reached the peak after rapid cooling-warming, which was more than twofold compared with CK (Fig. 3a). Adding SWCNTs could inhibit H_2_O_2_ content during the dehydration step, and H_2_O_2_ content kept at a low level in rapid cooling-warming and decreased significantly in dilution in the improved cryopreservation. O_2_^−^ inhibitation activities were higher in all steps of the control group, and OH· generation activities were higher after rapid cooling-warming in the SWCNTs-added cryopreservation (Fig. 3b, c). The MDA content trend was similar to that of H_2_O_2_. The MDA accumulation increased apparently during the control cryopreservation, and the EC treated with SWCNTs had less MDA than in control through the whole procedure (Fig. 3d).

### Effects of SWCNTs on the antioxidant system during cryopreservation

Adding SWCNTs significantly increased the activity of the antioxidant system during cryopreservation. With the addition of SWCNTs to the PVS2, the activities of enzymatic antioxidants highly increased, especially POD and CAT activities (Fig. 3f, g). The changes of SOD activity were not significant (Fig. 3e). The increases of POD activity treated by SWCNTs were much greater than those in the control process (Fig. 3f). Furthermore, the non-enzymatic antioxidant GSH in the SWCNTs group increased nearly twofold compared to the control group during dehydration (Fig. 3i).

### Correlation analysis of oxidative physiological indices

In the control cryopreservation (Table 1), MDA contents showed a significant positive correlation with H_2_O_2_ contents, which revealed that peroxidation was mainly caused by H_2_O_2_ in EC during cryopreservation. At the same time, OH· generation activities had a significant positive correlation with H_2_O_2_ and MDA contents. SOD activities had a significant positive correlation with CAT activities and AsA contents. In the SWCNTs cryopreservation (Table 2), O_2_^−^ inhibitation activities had a significant negative correlation with POD activities, MDA and AsA contents, and OH· generation activities had a significant positive correlation with SOD activities and AsA contents. MDA contents had a significant positive correlation with SOD activities, and SOD activities had a significant positive correlation with AsA contents like in the control cryopreservation. In addition, there was a significant positive correlation between POD and CAT activities indicating that the two enzymes may work together to decrease the ROS level during cryopreservation.

**Table 1:** The correlation analysis of physiological indices in the control cryopreservation

Indices	H_2_O_2_	O_2_^−^ inhibitation activity	OH· generation activity	MDA	SOD	POD	CAT	AsA	GSH
H_2_O_2_	1	0.263	0.918*	0.974**	0.630	− 0.141	0.510	0.702	0.830
O_2_^−^ inhibitation activity		1	− 0.076	0.146	− 0.477	0.381	− 0.678	− 0.319	0.190
OH· generation activity			1	0.888*	0.764	− 0.447	0.709	0.747	0.718
MDA				1	0.707	− 0.046	0.621	0.794	0.844
SOD					1	− 0.107	0.935*	0.973**	0.764
POD						1	− 0.277	0.075	0.269
CAT							1	0.878	0.527
AsA								1	0.859
GSH									1

All the data were correlation coefficients, and significant levels are indicated at * P < 0.05 or ** P < 0.01

**Table 2:** The correlation analysis of physiological indices in the SWCNTs-added cryopreservation

Indices	H_2_O_2_	O_2_^−^ inhibitation activity	OH· generation activity	MDA	SOD	POD	CAT	AsA	GSH
H_2_O_2_	1	− 0.388	0.042	0.440	0.195	0.451	0.490	0.166	0.529
O_2_^−^ inhibitation activity		1	− 0.793	− 0.904*	− 0.864	− 0.894*	− 0.867	− 0.884*	− 0.437
OH· generation activity			1	0.851	0.982**	0.568	0.550	0.979**	0.124
MDA				1	0.934*	0.868	0.875	0.877	0.147
SOD					1	0.693	0.683	0.980**	0.152
POD						1	0.996**	0.647	0.175
CAT							1	0.621	0.134
AsA								1	0.304
GSH									1

All the data were correlation coefficients, and significant levels are indicated at * P < 0.05 or ** P < 0.01

### Quantitative expression analysis of genes related to oxidative stress response

**Fig. 4:**
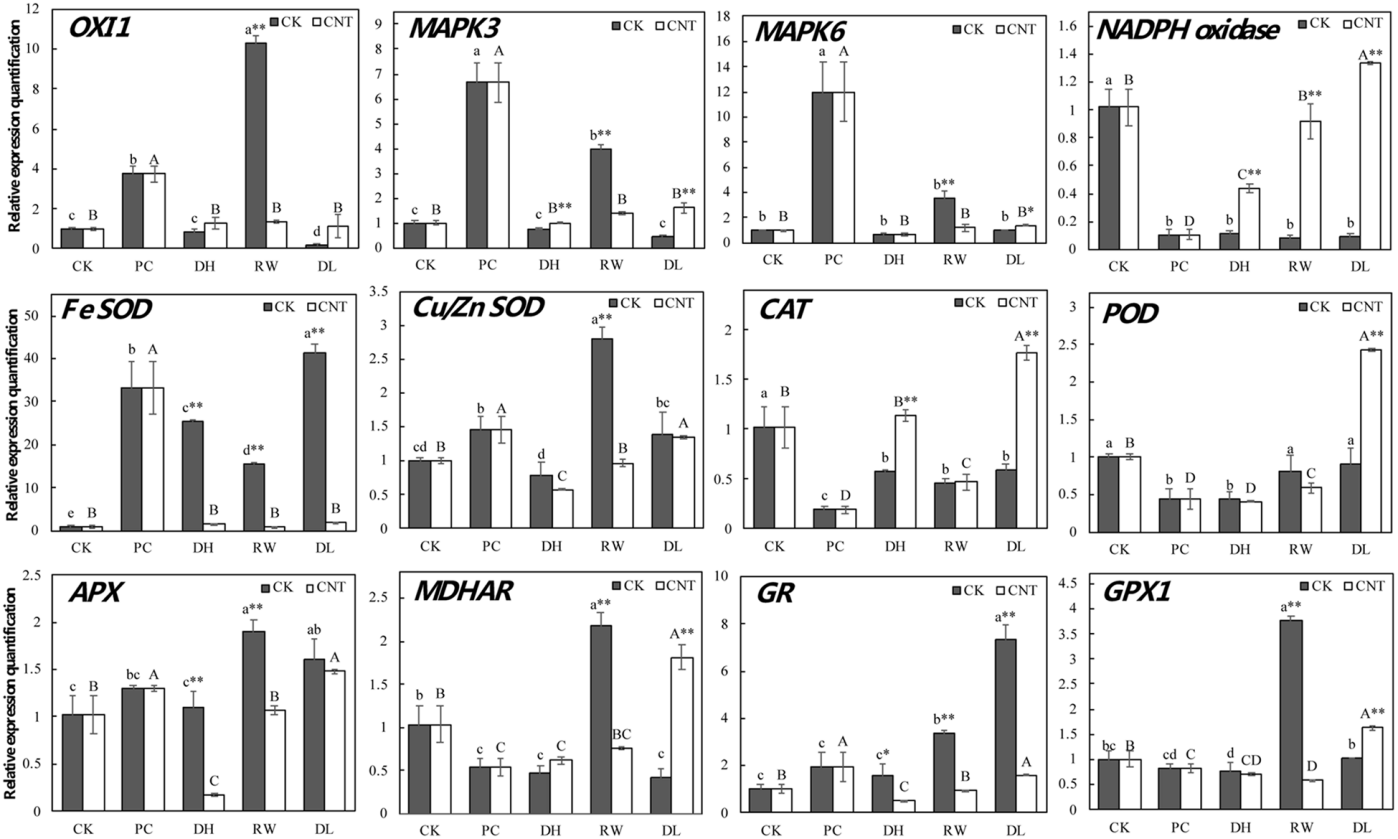
Real-time PCR quantitative analysis of ROS signal transduction and antioxidant system related genes between the control and the SWCNTs-added cryopreservation. Bars show means of gene expression level and standard deviation over triplicate detections. Values with different lowercase letters are significantly different among samples in the control group at 0.05 level. Values with different uppercase letters are significantly different among samples in the SWCNTs group at 0.05 level. * are significantly different between different groups in the same stage at 0.05 level, and ** are highly significantly different between different groups in the same stage at 0.01 level. The CK group is the cryopreservation without adding SWCNTs, and the CNT group is the SWCNTs-added cryopreservation. CK, untreated; PC, after pre-culture; DH, after dehydration; RW, after rapid cooling-warming; DL, after dilution

**Fig. 5:**
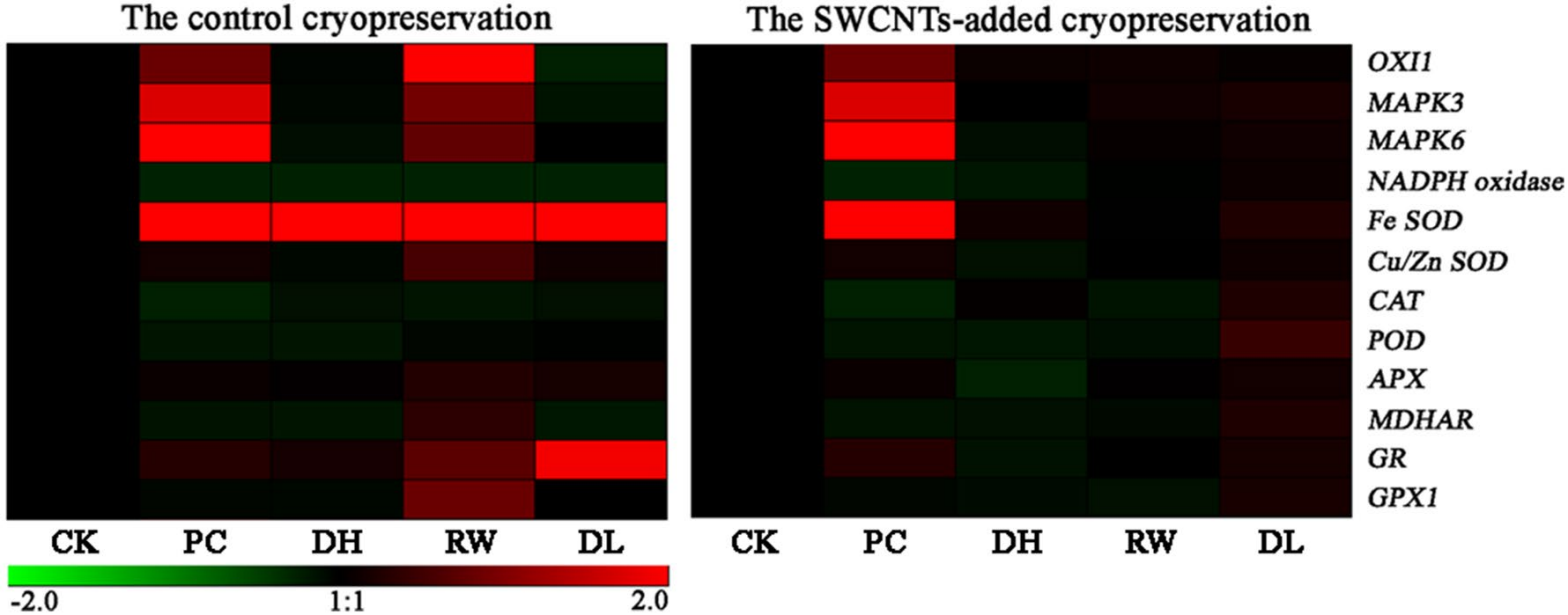
The heat-map of ROS signal transduction and antioxidant system related genes in the control and the SWCNTs-added cryopreservation. The up-regulated genes are indicated in red, and the down-regulated genes are indicated in green. The intensity of the colors increases as the expression differences increase, as shown in the bar at the bottom. CK, untreated; PC, after pre-culture; DH, after dehydration; RW, after rapid cooling-warming; DL, after dilution

Some genes related to oxidative stress response were chosen to study the molecular protection of SWCNTs during cryopreservation (Figs. 4, 5). As a ROS signal transduction related gene, *oxidative signal*-*inducible 1* (*OXI1*) was significantly upregulated at rapid cooling-warming stage in the control group, but maintained a low expression level in the SWCNTs group. *MAPK3*/*6* is located downstream of *OXI1*, and their expression patterns were very similar to that of *OXI1*. As a ROS signal amplification related gene, *NADPH oxidase* (*RbohA*) maintained a low expression level in the control group, and was significantly upregulated after dehydration in the SWCNTs group.

In the ROS-scavenging network, SOD and CAT catalyzed O_2_^−^ by forming H_2_O_2_ and H_2_O, respectively. The expression levels of *Cu/Zn* and *Fe SOD* were higher in the control group than SWCNTs group, but those of *CAT* were quite the opposite which shown that *CAT* was upregulated in SWCNTs group. The glutathione peroxidase (GPX) and AsA-GSH cycle were also essential to scavenge H_2_O_2_. Glutathione reductase (GR) and GPX drive the GPX cycle, and GR, monodehydroascorbate reductase (MDHAR), and ascorbate peroxidase (APX) regulate the AsA-GSH cycle. The expression levels of *GR* and *APX* were higher in the control group than SWCNTs group. The expression patterns of *GPX1* and *MDHAR* had the similar trend. They were only significantly upregulated in the rapid cooling-warming step of the control cryopreservation, and the dilution step of the SWCNTs cryopreservation. *POD* only significantly upregulated in the dilution step of the SWCNTs cryopreservation.

## Discussion

### SWCNTs contribute to the better efficiency of cryoprotectant

The field of plant cryobiology seeks to modify existing techniques that allow for the more efficient storage by reducing multiple stresses. Adding some exogenous compounds can improve the survival of cryopreserved cells in many species, like antioxidants [30, 36–39], anti-stress compounds [40, 41], metabolism related compounds [42, 43] and ice inhibitors [44, 45]. Previously we found that CNMs especially SWCNTs adding to PVS2 can improve the survival rate of *Arabidopsis* seedlings, callus or protocorm of *lily*, *Cymbidium* and *Anoectochilu* (unpublished results) and *A. praecox* callus [28] after cryopreservation. In this study, the relative survival rate was highly improved by adding SWCNTs to PVS2 in cryopreservation of *A. praecox* EC compared to the above compounds (Fig. 1). Thus, SWCNTs are the potential and important exogenous additions of cryoprotectant.

In our previous study, the differential scanning calorimetry (DSC) analysis suggested that the glass-transition temperature of SWCNTs-PVS2 slightly decreased [28]. Adding 0.1 g/L SWCNTs to PVS2 reduced the glass-transition temperature from − 112.67 to − 114.18 °C still within the reported temperature range of PVS2 (− 115 °C to − 112 °C). Moreover, a melting peak around − 38.87 °C and an endothermic peak at − 50.12 °C were observed in the DSC curve of 0.1 g/L SWCNTs-PVS2. It indicated that SWCNTs-PVS2 may be more stable than PVS2, but no significant changes on glass transition parameters were detected [28]. Since the glass-transition temperature is not significantly changed, do SWCNTs regulate the physiological response of plant cells?

### Effective antioxidant response during the dehydration step improved EC survival after cryopreservation

ROS-induced oxidative stress is a major reason of low survival in samples after cryopreservation [10, 46–53]. In many species cryopreservation, H_2_O_2_ is the major component of ROS leading to oxidative stress [8, 50]. Adding SWCNTs in the cryoprotectant might suppress H_2_O_2_ production and maintain the H_2_O_2_ content in the lower level than that in the control process, in which H_2_O_2_ content increased dramatically otherwise.

Membrane lipids are the primary target in oxidative damage [54], and MDA acting as a breakdown product of lipid peroxidation increased in cryopreserved *Oryza sativa* [52, 55], *Azadirachta indica* [56], *Arabidopsis thaliana* [8], *Agapanthus praecox* [10], *Hancornia speciose* [57], and *Passiflora suberosa* [53]. With the addition of NRSP in the cryoprotectant, the MDA content decreased and the SOD activity increased leading to more intact membrane and better quality of boar sperm [21]. The MDA content significantly increased in the control process, and led to the low survival rate of EC after cryopreservation.

The antioxidant system works to prevent plant cells from oxidative injury through cleaning ROS [11]. SWCNTs can activate related antioxidant enzymes, and improve survival after cryopreservation. In general, antioxidant enzyme activities increased after rapid cooling-warming (Fig. 3). In the SWCNTs cryopreservation, the enzyme activities rose up when cells were treated with SWCNTs-added PVS2, and maintained in a relative high level through the process. Like CAT was involved in scavenging the intracellular H_2_O_2_ in the SWCNTs-treated group, it is also found that cryopreserved of *Dendrobium* suffered serious oxidative stress because of decreasing CAT activity after cryopreservation leading to low survival [58], and high tolerance was related to high CAT activity in *Haematococcus pluvialis* cryopreservation [59]. In the study of Liu [60], the graphene-treated rice produced oxidative stress response, and the activities of SOD, POD and CAT were all increased in seedlings treated with graphene.

AsA-GSH cycle is a key way to scavenge H_2_O_2_ [61]. In this cycle, APX can scavenge H_2_O_2_ followed by a series of catalytic reactions involving GR, MDHAR and DHAR, in which GSH and AsA work as reducing substrates [62]. Adding SWCNTs increased GSH contents. Expression levels of *GPX* and *GR* in SWCNTs-added cryopreservation were lower than those in the control group, and the difference between these two processes became very obvious after rapid cooling-warming. This study indicated that PVS2 with SWCNTs improved the survival of *Agapanthus praecox*. SWCNTs promoted dehydration protection, and this improvement is due to the scavenging of ROS and improving of antioxidative system activity, especially POD and CAT.

### Effects of SWCNTs on ROS signal transduction during cryopreservation

Because of their key signaling roles (at low levels) and toxic roles (at high levels), the levels of ROS are regulated by the complex pathway including many genes [63–65]. In this pathway, plants sense ROS by three ways: (a) unknown ROS receptors; (b) redox-sensitive transcription factors; (c) direct inhibition of phosphatases [66]. The ROS signal is detected by unknown ROS receptors leading to the accumulation of Ca^2+^ signal [67–69], and the signal is transmitted to *oxidative signal*-*inducible 1* (*serine/threonine protein kinase*, *OXI1*) which was significantly upregulated in the control rapid cooling-warming with ROS level reaching the maximum which is much higher than that in the SWCNTs-added cryopreservation. OXI1 acts as a central factor in the ROS sensing, and is upregulated in many H_2_O_2_-generating stimulus [70]. *MAPK3/6* following *OXI1* [71, 72] were mainly upregulated during rapid cooling-warming of the control group.

The ROS signals further influenced following pathways including ROS producing and scavenging. The producing pathway has *NADPH oxidases* [73, 74], which were upregulated in the SWCNTs-treated steps. The producing pathway might be activated by ROS at low levels leading to the ROS production and amplification, and the scavenging pathway might be activated by ROS accumulation leading to the ROS suppression [66]. The interaction between the producing and scavenging pathway determines the intensity of ROS signals [66]. In summary, EC in the control group suffered excessive ROS after dehydration, which broke the ROS metabolism balance. By contrast, SWCNTs both enhanced the producing and scavenging pathway smoothly, and maintained ROS signals balanced and stable in EC during cryopreservation.

### Regulation of the physiological response by carbon nanotubes in plants

In the past decade, researchers have applied carbon nanotubes to plant studies and found that they have a certain regulatory effect on the physiological response, especially on enzyme activity and gene expression. The effective impact of CNMs on plant development and growth has been studied by many research groups [75]. Giraldo et al. [76] pointed out that SWCNTs localized in the lipid envelope of *Arabidopsis* chloroplasts, promoted over three times higher photosynthetic activity than that of controls, and concentrations of ROS inside chloroplasts were significantly suppressed. Industrialized MWCNTs can stimulate the growth of *Onobrychis arenaria* and enhance peroxidase activity [77]. In this study, SWCNTs contributed to the better efficiency of the cryoprotectant, and also improved enzyme activities including POD and CAT. At the molecular level, SWCNTs regulated gene expression levels including *NADPH oxidase*, *CAT* and *POD*. Other studies have found the similar results. The MWCNTs enhanced the tobacco cell growth, and upregulated genes related to water transport and cell division [78]. A number of genes regulated by MWCNTs were related to plant stress-signal transduction in tomato. Important stress signal pathways could be regulated in response to the uptake of carbon nanotubes [79]. For instance, *MAPK* was upregulated in leaves exposed to MWCNTs, and played a positive role in promoting plant development and stress response of carbon nanotubes [79]. In this study, *MAPK3/6* were higher in the control rapid cooling-warming than those treated with SWCNTs. Adding MWCNTs to the seeds of soybean (*Glycine max*), corn (*Zea mays*) and barley (*Hordeum vulgare*) led to the improvement of germination, and activated expression levels of aquaporins [80]. On the contrary, experiment on suspension rice cells with MWCNTs showed that it induced the accumulation of ROS leading to cell death [81]. It also decreases the dry weight and the SOD activity in *Arabidopsis* suspension cells [82]. Whether carbon nanotubes play a positive or negative regulatory role, they can regulate a variety of biological processes in plants, like water transport, cell division, stress response, electron transfer, ROS generation, and metabolism [83, 84]. However, the mechanism of how the nanotubes can regulate the physiological response especially regulate the gene expression is still an unresolved issue and deserved further studies.

## Conclusions

This study found that the relative survival rate was highly improved by adding SWCNTs to PVS2 in cryopreservation of *A. praecox* EC, and analyzed the oxidative response of EC at some steps in the control and SWCNTs-added cryopreservation. The SWCNTs entered EC at the step of dehydration, and mainly located around the cell wall and in the vesicles, and most of SWCNTs moved out of EC during the dilution step. The SWCNTs affected the ROS signal transduction pathway and antioxidant system response through the physiological and gene expression results. The EC treated with SWCNTs exhibited higher antioxidant levels, including POD, CAT and GSH than the EC in the control group. The EC mainly depended on the GPX and AsA-GSH cycle to scavenge H_2_O_2_ in the control cryopreservation, but depended on CAT in the SWCNTs-added cryopreservation which led to low levels of H_2_O_2_ and MDA. The elevated antioxidant level in dehydration by adding SWCNTs improved cells tolerance to injury from the cryopreservation procedure. The ROS signals of EC were balanced and stable in the SWCNTs-added cryopreservation. Overall, SWCNTs regulated the oxidative stress response of EC in cryopreservation and controlled cell oxidative injury by keeping ROS homeostasis to achieve a high survival rate after cryopreservation.

## Methods

### Plant material

The EC was induced and cultured from pedicel tissue of *A. praecox* as described by Wang et al. [34], and was maintained at 25 ± 2 °C in the dark by subculturing monthly onto the MS medium supplemented with 1.5 mg/L picloram. After 14 days of subculture, 0.2 g of EC was used as the replication for subsequent experiments.

### Experimental design

**Fig. 6:**
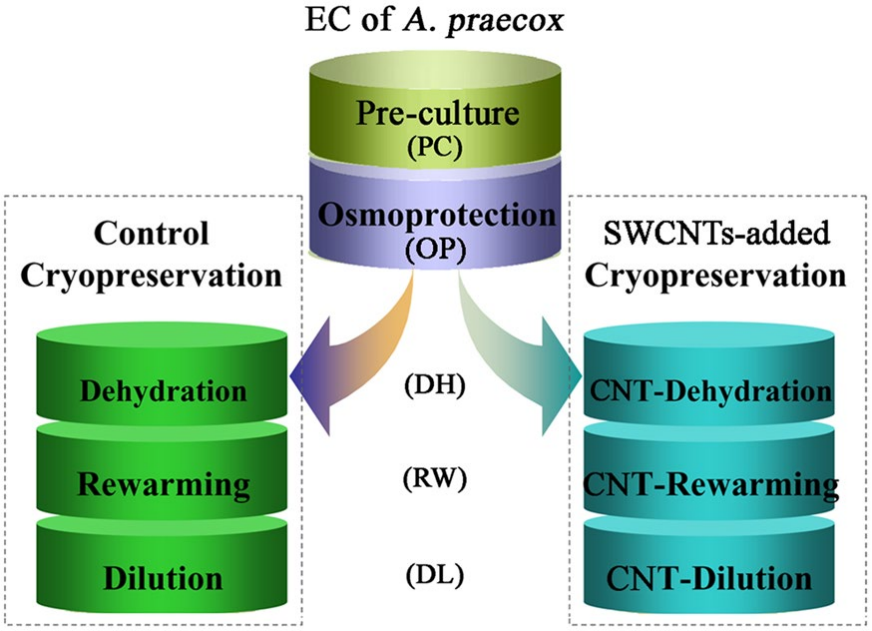
The samples in the study of SWCNTs to improve cryopreservation. The EC during the control and SWCNTs-added cryopreservation was taken as samples, including CK, after pre-culture, dehydration, rapid cooling-warming, and dilution

0.2 g of EC was cryopreserved as described by Chen et al. [30]. The cryopreservation procedure included pre-culture (PC), osmoprotection (OP), dehydration with PVS2 (DH), rapid cooling-warming (RW), dilution (DL), and recovery (RC) (Fig. 6). The SWCNTs aqueous solution (5.0 g/L, particle diameter 1 nm, length 1 μm) was kindly provided by Prof. Yafei Zhang (Shanghai Jiao Tong University, Shanghai, China). In the SWCNTs-added cryopreservation procedure, we added SWCNTs at 0.1 g/L in PVS2 at the dehydration step. The EC as samples for the physiological experiments and qRT-PCR analysis were taken after some key steps including CK, PC, DH, RW, and DL in the control and SWCNTs-added cryopreservation (Fig. 6). Experiments were performed three times individually.

### Viability detection

In order to detect the survival of cryopreserved EC, the 2,3,5-triphenyltetrazolium chloride (TTC) protocol was used in this study [30]. The EC (0.05 g) after 24 h-recovery was put into 2 mL TTC and kept in the dark for 20 h. After rinsing 3 times with sterile water, EC was immersed in 95% ethanol and 85 °C water bathed for 1 h. The EC was centrifuged at 3000*g* for 5 min, and the optical density value of supernatant was tested at 485 nm. The relative survival rate was the ratio of cryopreserved and non-cryopreserved value. Each sample procedure was repeated 3 times.

### Transmission electron microscopy

The EC (0.05 g) was treated as described by Chen et al. [28], and observed by 120 kV biology transmission electron microscope (Tecnai G2 spirit Biotwin).

### Detection of physiological indices

The EC (0.2 g) was used to detect the physiological indices. H_2_O_2_ levels, O_2_^−^ inhabitation and OH· generation activities, MDA content, SOD, CAT and POD activities, AsA and GSH contents were tested using the related biological assay kits (Nanjing Jiancheng Bioengineering Institute, China) following the manufacturer’s instructions with some modifications according to Yang et al. [85].

### qRT-PCR analysis

The qRT-PCR was performed as described by Chen et al. [30]. The EC (0.1 g) was collected for total RNA extraction using MiniBEST plant RNA extraction kit (TaKaRa, Otsu, Shiga, Japan) according to the manufacturer’s instructions. The amplifications were repeated for 3 times. The relative quantitative expression was calculated using the 2^−△△CT^ method. The *actin* was used as an internal control parameter for normalization, and all primer sequences were listed in Additional file 1: Table S1 including *APX*, *CAT*, *Fe SOD*, *Cu/Zn SOD*, *POD*, *MDHAR*, *GPX1*, *GR*, *NADPH oxidase*, *OXI1*, *MAPK3*, and *MAPK6*. The heat-map figures were made by GENESIS Software after the normalization of gene expression data.

### Statistical analysis

The one-way ANOVA was used to analyze differences followed by the least significant difference multiple range test using Statistics Analysis System 9.1.3 software (SAS Institute, Inc., Cary, NC, USA). Correlation analysis was calculated by Statistics Analysis System 9.1.3 software, and P < 0.05 was considered as significant.

## Supplementary information

**Additional file 1: Table S1.** Primers sequences of qRT-PCR.

## Data Availability

All data generated or analysed during this study are included in this published article and its Additional file 1: Table S1.

## Abbreviations

ABA: Abscisic acid
APX: Ascorbate peroxidase
AsA: Ascorbic acid
CAT: Catalase
CNMs: Carbon nanomaterials
CNTs: Carbon nanotubes
CW: Cell wall
DH: Dehydration
DHAR: Dehydroascorbate reductase
DL: Dilution
DSC: Differential scanning calorimetry
EC: Embryogenic callus
ER: Endoplasmic reticulum
G: Golgi apparatus
GPX: Glutathione peroxidase
GR: glutathione reductase
GSH: Glutathione
H_2_O_2_: Hydrogen peroxide
LA: Lipoic acid
LB: Lipid body
LN: Liquid nitrogen
LSD: Least significant difference
M: Mitochondrion
MAPK: Mitogen-activated protein kinase
MDA: Malonaldehyde
MDHAR: Monodehydroascorbate reductase
MS: Murashige and Skoog
NRSP: Nanomaterials Rhodiola Sachanensis Polysaccharide
O_2_^−^: Superoxide anion
OH: Hydroxyl radicals
OP: Osmoprotection
OXI1: Oxidative signal-inducible 1
PC: Pre-culture
PVA: polyvinyl alcohol
PVP: Polyvinyl pyrrolidone
PVS: Plant vitrification solution
qRT-PCR: Quantitative reverse-transcription polymerase chain reactions
RC: Recovery
ROS: Reactive oxygen species
RW: Rapid cooling-warming
SG: Starch grains
SOD: Superoxide dismutase
SWCNTs: Single-wall carbon nanotubes
TEM: Transmission electron microscopy
TTC: Triphenyltetrazolium chloride
VES: Vesicle
