# Supplementary material for: Single-wall carbon nanotubes improve cell survival rate and reduce oxidative injury in cryopreservation of Agapanthus praecox embryogenic callus
Source: Plant Methods. 2020 Sep 21;16:130. doi: 10.1186/s13007-020-00674-6 (PMC7507619; doi:10.1186/s13007-020-00674-6)
Supplement: Supplementary file 1 — Additional file 1: Table S1. Primers sequences of qRT-PCR. [file 13007_2020_674_MOESM1_ESM.docx]

**Additional file 1: Table S1.** Primers sequences of qRT-PCR

| Abbreviation | Gene name | Sense and Anti-sense Primer (5' to 3') |
| --- | --- | --- |
| *ACTIN* | *ACTIN* | 5’-CAGTGTCTGGATTGGAGG-3’ |
|  |  | 5’-TAGAAGCACTTCCTGTG-3’ |
| *APX* | *L-ascorbate peroxidase* | 5’-CTAAGCGGAGCATCAAGG-3’ |
|  |  | 5’-AACAGTGAGCGAGGAGTA-3’ |
| *CAT* | *Catalase* | 5’-TCGTGGATAACAGTGGAG-3’ |
|  |  | 5’-AGGACTACCATCTCATCG-3’ |
| *Fe SOD* | *Superoxide dismutase [Fe]* | 5’-CATCCCATCAAGACGAAACT-3’ |
|  |  | 5’-GAGTGAAGCAAGACGAGAG-3’ |
| *Cu/Zn SOD* | *Superoxide dismutase [Cu-Zn]* | 5’-AATCGCTGAGGCAACTAT-3’ |
|  |  | 5’-ATGAACCACAAATGCTCTC-3’ |
| *POD* | *Peroxidase* | 5’-ACAAGAGGCACAAGAACA-3’ |
|  |  | 5’-TGAATCCAGCAGCAATGA-3’ |
| *MDHAR* | *Monodehydroascorbate reductase (NADH)* | 5’-CTTCTTCGCTTGTATTGTTG-3’ |
|  |  | 5’-GTCTCATCATTGCTACTGG-3’ |
| *GPX1* | *Glutathione peroxidase 1* | 5’-GATGCGGCTGATTGAGAT-3’ |
|  |  | 5’-CTTGCTCTTCCTCTGCTT-3’ |
| *GR* | *Glutathione reductase* | 5’-TTGTGTTCTTCGTGGATG-3’ |
|  |  | 5’-TTAGGCTCAGTCTCATAGTT-3’ |
| *NADPH oxidase* | *Respiratory burst oxidase* | 5’-GGCATCCATTCTCCATTAC-3’ |
|  |  | 5’-GTCCACTTCTTCCATTCATT-3’ |
| *OXI1* | *Serine/threonine-protein kinase OXI1* | 5’-TCGCCGAAGGAATACAAG-3’ |
|  |  | 5’-ACATTTGGGTGGTCTATCAA-3’ |
| *MAPK3* | *Mitogen-activated protein kinase 3* | 5’-ACGAAGAATTGGAAGAAGGA-3’ |
|  |  | 5’-AACCACAAGCACCATACA-3’ |
| *MAPK6* | *Mitogen-activated protein kinase 6* | 5’-TGTAGCCGAAGGAATCATAA-3’ |
|  |  | 5’-GTAAGAGCGAAGAAGATAACG-3’ |
